# Supplementary material for: Key Pathways and Genes of Arabidopsis thaliana and Arabidopsis halleri Roots under Cadmium Stress Responses: Differences and Similarities
Source: Plants (Basel). 2023 Apr 27;12(9):1793. doi: 10.3390/plants12091793 (PMC10180823; doi:10.3390/plants12091793)
Supplement: Supplementary file 1 [file plants-12-01793-s001.zip › Supplementary figures.pdf]

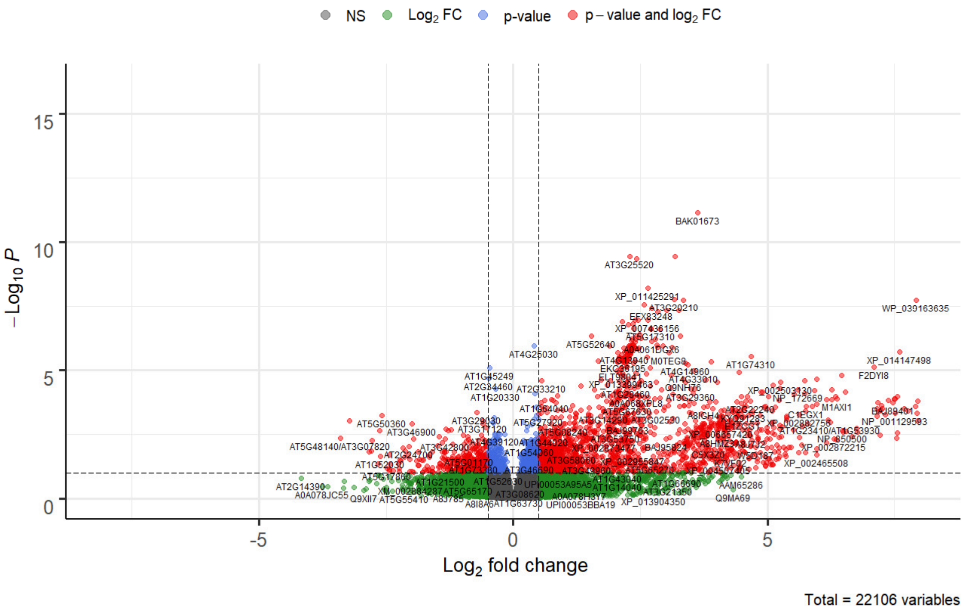

Figure S1.Volcanoplot of the up- and down- regulated genes in A. halleri. The volcano plot shows significantly (adjusted p value <0.05) upregulated and downregulated genes (DEGs) of A. halleri root under Cd stress with a fold change higher than 0.5 (red dots). Dashed horizontal line for significance threshold, vertical dashed lines for log_2 fold change (FC) threshold.

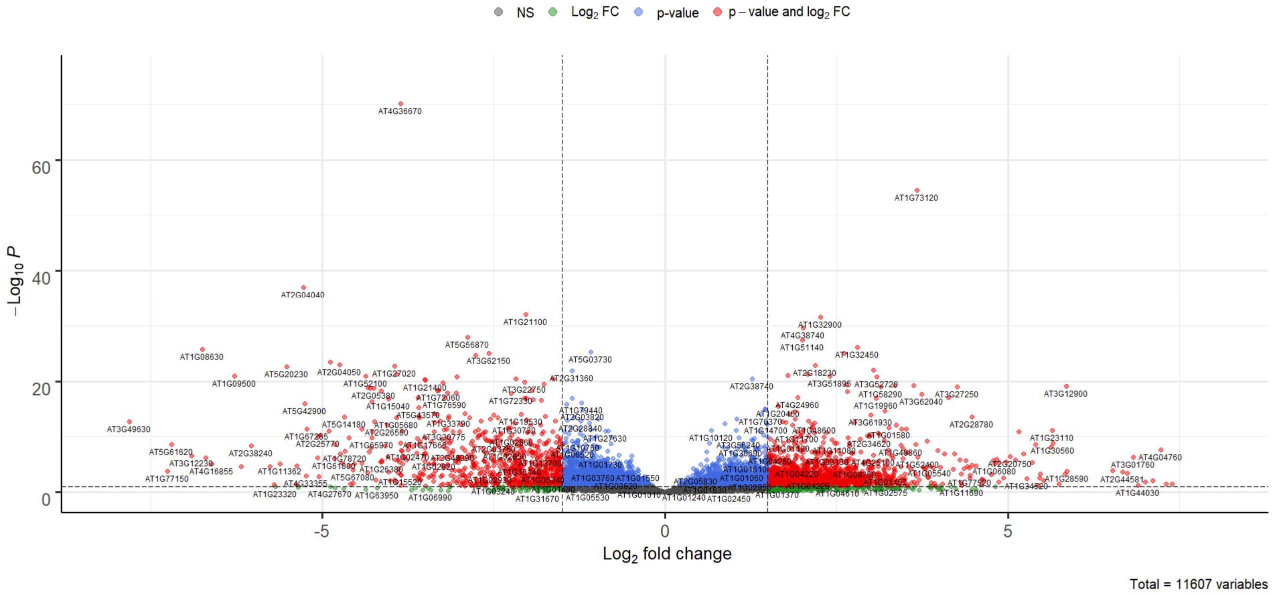

Figure S2. Volcanoplot of the up- and down- regulated genes in A. thaliana. The volcano plot shows significantly (adjusted p value <0.05) upregulated and downregulated genes (DEGs) of A. thaliana root under Cd stress with a fold change higher than 0.5 (red dots). Dashed horizontal line for significance threshold, vertical dashed lines for log_2 fold change (FC) threshold.
